# Supplementary material for: Safety, Immunogenicity and Lot-to-Lot Consistency of Sabin-Strain Inactivated Poliovirus Vaccine in 2-Month-Old Infants: A Double-Blind, Randomized Phase III Trial
Source: Vaccines (Basel). 2022 Feb 8;10(2):254. doi: 10.3390/vaccines10020254 (PMC8879689; doi:10.3390/vaccines10020254)
Supplement: Supplementary file 1 [file vaccines-10-00254-s001.zip › vaccines-1564104-supplementary.pdf]

Table S1. Summary of adverse events after vaccination

Adverse events	sIPV 1 n (%)	sIPV 2 n (%)	sIPV 3 n (%)	Pooled sIPV n (%)	IPV	*P*-value[a]	*P*-value[b]
**Overall**	225 (69.23)	207 (63.69)	225 (69.23)	657 (67.38)	216 (66.46)	0.2319	0.7851
Dose1 (N=325/325/325)	134 (41.23)	115 (35.38)	130 (40.00)	379 (38.87)	123 (37.85)	0.2773	0.7925
Dose 2(N=320/323/316)	116 (36.25)	119 (36.84)	111 (35.13)	346 (36.08)	101 (32.37)	0.9042	0.2464
Dose 3(N=317/321/310)	99 (31.23)	90 (28.04)	119 (38.39)	308 (32.49)	102 (32.80)	0.0188	0.9444
Solicited	132 (40.62)	127 (39.08)	134 (41.23)	393 (40.31)	119 (36.62)	0.8418	0.2651
Unsolicited	162 (49.85)	143 (44.00)	153 (47.08)	458 (46.97)	152 (46.77)	0.3289	1.0000
Within 30 min	25 (7.69)	38 (11.69)	26 (8.00)	89 (9.13)	25 (7.69)	0.1591	0.4971
0-7 days	151 (46.46)	146 (44.92)	158 (48.62)	455 (46.67)	145 (44.62)	0.6448	0.5632
8-30 days	126 (38.77)	123 (37.85)	129 (39.69)	378 (38.77)	125 (38.46)	0.8953	0.9476
0-30 days	222 (68.31	207 (63.69)	223 (68.62)	652 (66.87)	214 (65.85)	0.3402	0.7346
After 30 days	17 (5.23)	6 (1.85)	9 (2.77)	32 (3.28)	13 (4.00)	0.0536	0.5989
**Unrelated**	158 (48.62)	142 (43.69)	145 (44.62)	445 (45.64)	153 (47.08)	0.4146	0.6535
**Related**	135 (41.54)	129 (39.69)	140 (43.08)	404 (41.44)	121 (37.23)	0.6862	0.1920
Dose 1(N=325/325/325)	85 (26.15)	65 (20.00)	78(24.00)	228 (23.38)	71 (21.85)	0.1712	0.5948
Dose 2(N=320/323/316)	55 (17.19)	60 (18.58)	52 (16.46)	167 (17.41)	40 (12.82)	0.7781	0.0636
Dose 3(N=317/321/310)	45 (14.20)	45 (14.02)	53 (17.10)	143 (15.08)	37 (11.90)	0.4869	0.1910
Solicited	130 (40.00)	125 (38.46)	132 (40.62)	387 (39.69)	118 (36.31)	0.8411	0.2934
Systemic	115 (35.38)	101 (31.08)	116 (35.69)	332 (34.05)	95 (29.23)	0.3867	0.1169
Local	27 (8.31)	39 (12.00)	25 (7.69)	91 (9.33)	30 (9.23)	0.1420	1.0000
Unsolicited	13 (4.00)	6 (1.85)	17 (5.23)	36 (3.69)	9 (2.77)	0.0625	0.4882

Adverse events	sIPV 1<br>n (%)	sIPV 2<br>n (%)	sIPV 3<br>n (%)	Pooled sIPV<br>n (%)	IPV	***P*-value[a]**	***P*-value[b]**
Within 30 min	25 (7.69)	38 (11.69)	25 (7.69)	88 (9.03)	25 (7.69)	0.1460	0.4970
0-7 days	135 (41.54)	129 (39.69)	139 (42.77)	403 (41.33)	120 (36.92)	0.7305	0.1704
8-30 days	1 (0.31)	0 (0.00)	1 (0.31)	2 (0.21)	1 (0.31)	1.0000	1.0000
0-30 days	135 (41.54)	129 (39.69)	140 (43.08)	404 (41.44)	121 (37.23)	0.6862	0.1920

Table S2 The severity of adverse reactions among different groups

Adverse reactions	sIPV 1 n (%)	sIPV 2 n (%)	sIPV 3 n (%)	Pooled sIPV n (%)	IPV n (%)	*P* value [a]	*P* value [b]
**Overall**	135(41.54)	129(39.69)	140(43.08)	404(41.44)	121(37.23)	0.6862	0.1920
Grade 1	120(36.92)	114(35.08)	114(35.08)	348(35.69)	99(30.46)	0.8724	0.0919
Grade 2	42(12.92)	33(10.15)	54(16.62)	129(13.23)	42(12.92)	0.0541	0.9248
Grade 3	3(0.92)	2(0.62)	3(0.92)	8(0.82)	2(0.62)	1.0000	1.0000
**General disorders and administration site conditions**	92(28.31)	97(29.85)	96(29.54)	285(29.23)	86(26.46)	0.9030	0.3571
Grade 1	79(24.31)	84(25.85)	71(21.85)	234(24)	62(19.08)	0.4826	0.0673
Grade 2	25(7.69)	22(6.77)	36(11.08)	83(8.51)	26(8)	0.1233	0.8182
Grade 3	1(0.31)	2(0.62)	3(0.92)	6(0.62)	2(0.62)	0.8758	1.0000
Fever	66(20.31)	61(18.77)	74(22.77)	201(20.62)	54(16.62)	0.4579	0.1256
Grade 1	47(14.46)	48(14.77)	50(15.38)	145(14.87)	31(9.54)	0.9608	0.0148
Grade 2	22(6.77)	18(5.54)	32(9.85)	72(7.38)	22(6.77)	0.1097	0.8049
Grade 3	0(0)	2(0.62)	3(0.92)	5(0.51)	1(0.31)	0.3809	1.0000
Injection site erythema	25(7.69)	36(11.08)	19(5.85)	80(8.21)	27(8.31)	0.0574	1.0000
Grade 1	25(7.69)	34(10.46)	17(5.23)	76(7.79)	25(7.69)	0.0469	1.0000
Grade 2	0(0)	2(0.62)	2(0.62)	4(0.41)	4(1.23)	0.5542	0.1131
Irritability postvaccinal	17(5.23)	7(2.15)	4(1.23)	28(2.87)	6(1.85)	0.0092	0.4224
Grade 1	16(4.92)	6(1.85)	3(0.92)	25(2.56)	5(1.54)	0.0049	0.3937
Grade 2	4(1.23)	1(0.31)	1(0.31)	6(0.62)	0(0)	0.3804	0.3463
Grade 3	1(0.31)	0(0)	0(0)	1(0.1)	1(0.31)	1.0000	0.4376
Vaccination site pain	1(0.31)	3(0.92)	4(1.23)	8(0.82)	0(0)	0.5493	0.2125
Grade 1	1(0.31)	3(0.92)	2(0.62)	6(0.62)	0(0)	0.8758	0.3463

Adverse reactions	sIPV 1 n (%)	sIPV 2 n (%)	sIPV 3 n (%)	Pooled sIPV n (%)	IPV n (%)	*P* value [a]	*P* value [b]
Grade 2	0(0)	0(0)	2(0.62)	2(0.21)	0(0)	0.3326	1.0000
Vaccination site swelling	1(0.31)	3(0.92)	1(0.31)	5(0.51)	0(0)	0.6281	0.3398
Grade 1	1(0.31)	1(0.31)	1(0.31)	3(0.31)	0(0)	1.0000	0.5776
Grade 2	0(0)	2(0.62)	0(0)	2(0.21)	0(0)	0.3326	1.0000
Injection site induration	0(0)	2(0.62)	3(0.92)	5(0.51)	1(0.31)	0.3809	1.0000
Grade 1	0(0)	1(0.31)	3(0.92)	4(0.41)	1(0.31)	0.3320	1.0000
Grade 2	0(0)	1(0.31)	1(0.31)	2(0.21)	0(0)	1.0000	1.0000
Vaccination site rash	0(0)	0(0)	2(0.62)	2(0.21)	2(0.62)	0.3326	0.2616
Grade 1	0(0)	0(0)	2(0.62)	2(0.21)	2(0.62)	0.3326	0.2616
Vaccination site pruritus	1(0.31)	0(0)	1(0.31)	2(0.21)	1(0.31)	1.0000	1.0000
Grade 1	1(0.31)	0(0)	1(0.31)	2(0.21)	1(0.31)	1.0000	1.0000
**Gastrointestinal disorders**	50(15.38)	42(12.92)	56(17.23)	148(15.18)	56(17.23)	0.3183	0.3795
Grade 1	40(12.31)	35(10.77)	50(15.38)	125(12.82)	46(14.15)	0.2131	0.5697
Grade 2	11(3.38)	10(3.08)	12(3.69)	33(3.38)	16(4.92)	0.9746	0.2381
Grade 3	2(0.62)	0(0)	0(0)	2(0.21)	0(0)	0.3326	1.0000
Diarrhoea	35(10.77)	30(9.23)	35(10.77)	100(10.26)	33(10.15)	0.7805	1.0000
Grade 1	29(8.92)	25(7.69)	32(9.85)	86(8.82)	22(6.77)	0.6437	0.2961
Grade 2	5(1.54)	7(2.15)	4(1.23)	16(1.64)	12(3.69)	0.7385	0.0438
Grade 3	2(0.62)	0(0)	0(0)	2(0.21)	0(0)	0.3326	1.0000
Vomiting	21(6.46)	18(5.54)	26(8)	65(6.67)	27(8.31)	0.4807	0.3191
Grade 1	14(4.31)	15(4.62)	22(6.77)	51(5.23)	25(7.69)	0.3532	0.1031
Grade 2	7(2.15)	3(0.92)	7(2.15)	17(1.74)	3(0.92)	0.4150	0.4358
Constipation	1(0.31)	0(0)	1(0.31)	2(0.21)	0(0)	1.0000	1.0000

Adverse reactions	sIPV 1<br>n (%)	sIPV 2<br>n (%)	sIPV 3<br>n (%)	Pooled sIPV<br>n (%)	IPV<br>n (%)	*P* value [a]	*P* value [b]
Grade 1	0(0)	0(0)	1(0.31)	1(0.1)	0(0)	1.0000	1.0000
Grade 2	1(0.31)	0(0)	0(0)	1(0.1)	0(0)	1.0000	1.0000
Dyspepsia	0(0)	0(0)	1(0.31)	1(0.1)	4(1.23)	1.0000	0.0154
Grade 1	0(0)	0(0)	1(0.31)	1(0.1)	3(0.92)	1.0000	0.0505
Grade 2	0(0)	0(0)	0(0)	0(0)	1(0.31)	1.0000	0.2500
Faeces discoloured	0(0)	0(0)	0(0)	0(0)	1(0.31)	1.0000	0.2500
Grade 1	0(0)	0(0)	0(0)	0(0)	1(0.31)	1.0000	0.2500
Abdominal distension	0(0)	1(0.31)	0(0)	1(0.1)	0(0)	1.0000	1.0000
Grade 2	0(0)	1(0.31)	0(0)	1(0.1)	0(0)	1.0000	1.0000
Gagging	0(0)	0(0)	1(0.31)	1(0.1)	0(0)	1.0000	1.0000
Grade 2	0(0)	0(0)	1(0.31)	1(0.1)	0(0)	1.0000	1.0000
**Appetite and general nutritional disorders**	19(5.85)	9(2.77)	5(1.54)	33(3.38)	12(3.69)	0.0096	0.8609
Grade 1	13(4)	8(2.46)	3(0.92)	24(2.46)	10(3.08)	0.0414	0.5492
Grade 2	8(2.46)	2(0.62)	2(0.62)	12(1.23)	2(0.62)	0.0682	0.5373
Decreased appetite	19(5.85)	9(2.77)	5(1.54)	33(3.38)	12(3.69)	0.0096	0.8609
Grade 1	13(4)	8(2.46)	3(0.92)	24(2.46)	10(3.08)	0.0414	0.5492
Grade 2	8(2.46)	2(0.62)	2(0.62)	12(1.23)	2(0.62)	0.0682	0.5373
**Respiratory, thoracic and mediastinal disorders**	12(3.69)	5(1.54)	14(4.31)	31(3.18)	4(1.23)	0.0981	0.0734
Grade 1	6(1.85)	5(1.54)	7(2.15)	18(1.85)	3(0.92)	0.9549	0.3176
Grade 2	6(1.85)	1(0.31)	7(2.15)	14(1.44)	1(0.31)	0.1076	0.1345
Runny nose	6(1.85)	5(1.54)	8(2.46)	19(1.95)	3(0.92)	0.7729	0.3198
Grade 1	5(1.54)	5(1.54)	4(1.23)	14(1.44)	2(0.62)	1.0000	0.3838

Adverse reactions	sIPV 1 n (%)	sIPV 2 n (%)	sIPV 3 n (%)	Pooled sIPV n (%)	IPV n (%)	*P* value [a]	*P* value [b]
Grade 2	1(0.31)	0(0)	4(1.23)	5(0.51)	1(0.31)	0.1345	1.0000
Cough	3(0.92)	2(0.62)	4(1.23)	9(0.92)	4(1.23)	0.9139	0.7472
Grade 1	1(0.31)	1(0.31)	3(0.92)	5(0.51)	3(0.92)	0.6281	0.4206
Grade 2	2(0.62)	1(0.31)	1(0.31)	4(0.41)	1(0.31)	1.0000	1.0000
Nasal obstruction	3(0.92)	0(0)	2(0.62)	5(0.51)	0(0)	0.3809	0.3398
Grade 1	1(0.31)	0(0)	0(0)	1(0.1)	0(0)	1.0000	1.0000
Grade 2	2(0.62)	0(0)	2(0.62)	4(0.41)	0(0)	0.5542	0.5775
Nasal congestion	1(0.31)	0(0)	0(0)	1(0.1)	0(0)	1.0000	1.0000
Grade 2	1(0.31)	0(0)	0(0)	1(0.1)	0(0)	1.0000	1.0000
Oropharyngeal pain	0(0)	0(0)	1(0.31)	1(0.1)	0(0)	1.0000	1.0000
Grade 1	0(0)	0(0)	1(0.31)	1(0.1)	0(0)	1.0000	1.0000
Pharyngeal erythema	0(0)	0(0)	1(0.31)	1(0.1)	0(0)	1.0000	1.0000
Grade 2	0(0)	0(0)	1(0.31)	1(0.1)	0(0)	1.0000	1.0000
**Skin and subcutaneous tissue disorders**	6(1.85)	7(2.15)	6(1.85)	19(1.95)	3(0.92)	1.0000	0.3198
Grade 1	5(1.54)	5(1.54)	4(1.23)	14(1.44)	2(0.62)	1.0000	0.3838
Grade 2	1(0.31)	2(0.62)	2(0.62)	5(0.51)	1(0.31)	1.0000	1.0000
Mucocutaneous rash	6(1.85)	7(2.15)	6(1.85)	19(1.95)	1(0.31)	1.0000	0.0368
Grade 1	5(1.54)	5(1.54)	4(1.23)	14(1.44)	1(0.31)	1.0000	0.1345
Grade 2	1(0.31)	2(0.62)	2(0.62)	5(0.51)	0(0)	1.0000	0.3398
Haemorrhage subcutaneous	0(0)	0(0)	0(0)	0(0)	1(0.31)	1.0000	0.25
Grade 1	0(0)	0(0)	0(0)	0(0)	1(0.31)	1.0000	0.25
Rash	0(0)	0(0)	0(0)	0(0)	1(0.31)	1.0000	0.2500

Adverse reactions	sIPV 1<br>n (%)	sIPV 2<br>n (%)	sIPV 3<br>n (%)	Pooled sIPV<br>n (%)	IPV<br>n (%)	*P* value [a]	*P* value [b]
Grade 2	0(0)	0(0)	0(0)	0(0)	1(0.31)	1.0000	0.2500
**Nervous system disorders**	7(2.15)	2(0.62)	3(0.92)	12(1.23)	7(2.15)	0.2628	0.2829
Grade 1	5(1.54)	2(0.62)	3(0.92)	10(1.03)	5(1.54)	0.6236	0.5474
Grade 2	3(0.92)	1(0.31)	0(0)	4(0.41)	2(0.62)	0.332	0.6436
Seizure	5(1.54)	1(0.31)	2(0.62)	8(0.82)	4(1.23)	0.2929	0.508
Grade 1	3(0.92)	1(0.31)	2(0.62)	6(0.62)	3(0.92)	0.8758	0.6987
Grade 2	2(0.62)	0(0)	0(0)	2(0.21)	1(0.31)	0.3326	1.0000
Depressed level of consciousness	4(1.23)	1(0.31)	1(0.31)	6(0.62)	3(0.92)	0.3804	0.6987
Grade 1	3(0.92)	1(0.31)	1(0.31)	5(0.51)	2(0.62)	0.6281	1.0000
Grade 2	1(0.31)	1(0.31)	0(0)	2(0.21)	1(0.31)	1.0000	1.0000
**Immune system disorders**	1(0.31)	4(1.23)	0(0)	5(0.51)	0(0)	0.1345	0.3398
Grade 1	0(0)	4(1.23)	0(0)	4(0.41)	0(0)	0.0366	0.5775
Grade 2	1(0.31)	0(0)	0(0)	1(0.1)	0(0)	1.0000	1.0000
Hypersensitivity	1(0.31)	4(1.23)	0(0)	5(0.51)	0(0)	0.1345	0.3398
Grade 1	0(0)	4(1.23)	0(0)	4(0.41)	0(0)	0.0366	0.5775
Grade 2	1(0.31)	0(0)	0(0)	1(0.1)	0(0)	1.0000	1.0000
